# Automation of Presentation Record Production Based on Rich-Media Technology Using SNT Petri Nets Theory

**DOI:** 10.1155/2015/303705

**Published:** 2015-07-14

**Authors:** Ivo Martiník

**Affiliations:** Faculty of Economics, VŠB-Technical University of Ostrava, Sokolská třída 33, 701 21 Ostrava 1, Czech Republic

## Abstract

Rich-media describes a broad range of digital interactive media that is increasingly used in the Internet and also in the support of education. Last year, a special pilot audiovisual lecture room was built as a part of the MERLINGO (*MEdia-rich Repository of LearnING Objects*) project solution. It contains all the elements of the modern lecture room determined for the implementation of presentation recordings based on the rich-media technologies and their publication online or on-demand featuring the access of all its elements in the automated mode including automatic editing. Property-preserving Petri net process algebras (PPPA) were designed for the specification and verification of the Petri net processes. PPPA does not need to verify the composition of the Petri net processes because all their algebraic operators preserve the specified set of the properties. These original PPPA are significantly generalized for the newly introduced class of the SNT Petri process and agent nets in this paper. The PLACE-SUBST and ASYNC-PROC algebraic operators are defined for this class of Petri nets and their chosen properties are proved. The SNT Petri process and agent nets theory were significantly applied at the design, verification, and implementation of the programming system ensuring the pilot audiovisual lecture room functionality.

## 1. Introduction

The term rich-media describes a broad range of digital interactive media, through which it is possible to share and transfer information and communicate in various ways. Moreover, rich-media enable interactivity, that is, bidirectional communication. The characteristic feature of the rich-media technologies is their accessibility online or on-demand, followed by the support of the dynamics of changes. An example can be online streaming video reporting, which is updated during broadcast, or a record of presentation placed on a website jointly with the synchronized slide show, which the user can interactively work with. Currently, there are several theories dealing with various aspects of the rich-media implementation, such as* Media Richness Theory* [1],* Media Naturalness Theory* [2], and* Social Presence Theory* [3].

The issue of majority aspects in implementing rich-media technologies at selected universities in the Czech Republic is dealt with in the MERLINGO project (*MEdia-rich Repository of LearnING Objects*) [4]. One of the key objectives of this project last year was the implementation of the cutting edge rich-media technologies in order to build a specialized audiovisual lecture room containing all the elements of a modern lecture room determined for the implementation of presentation recordings and their publication online or on-demand featuring the integrated ergonomic and intuitive control and access in the automated mode of all its elements including automatic editing. Next objective was to provide a possibility of parallel sharing and feedback of lectures presented in the lecture room outfitted as such in real time even at other audiovisual auditoriums while providing the services of the central repository of educational objects MERLINGO and the possibility of their adaptation for the students with special needs. The implementation of those functionalities significantly contributed in decrease of costs required for the operation of high-capacity lecture halls including reduction of time and space demands mainly required for teaching subjects determined for large groups of students. Currently, the operation of the central repository MERLINGO dramatically simplifies the implementation of the new revolutionary technologies* EduArt* [5] and* MediaInTouch* [6], featuring unique characteristics in this area complying with demanding requirements of teachers in the availability and quality of presentation recordings.

Petri nets [7–14] represent a popular formalism connecting advantages of the graphic representation of a modeled system with the possibilities of its simulation and the formal analyzability. Property-preserving Petri net process algebras (PPPA) [15] were originally designed for the specification and verification of manufacturing systems. PPPA also follows many ideas that can be originally seen in the class of workflow nets [16]. The elements of PPPA are the Petri net processes, ordinary connected Petri nets with a unique entry place, a unique exit place, and a set of places for handling resource sharing with their initial marking allowed. Among other features, PPPA does not need to verify composite components because all its operators preserve many properties. Hence, if the primitive modules satisfy the desirable properties, each of the composite components, including the system itself, also satisfies these properties.

PPPA has five types of operators: extensions, compositions, refinements, reductions, and place-merging. All the operators can preserve about twenty properties (some under additional conditions), such as liveliness, boundedness, reversibility, traps, siphons, and proper termination.

The main goals of this paper are to generalize PPPA for the newly introduced class of the SNT Petri process and agent nets (SNTPAN) and to define the new SNTPAN operators called PLACE-SUBST (*PLACE-SUBSTitution*) and ASYNC-PROC (*ASYNChronous-PROCessing*) with the property of the SNTPAN soundness preservation. This class of SNTPANs with the support of the PLACE-SUBST and ASYNC-PROC operators that preserve the soundness property during the SNTPAN components composition process has been applied at the design, verification, simulation, and implementation phases of the software support of the audiovisual lecture room enabling automated recordings of the presentations including automated editing.

## 2. Materials and Methods

### 2.1. SNT Petri Process and Agent Nets and Their Properties

Let **N** denote the set of all natural numbers, **N**_0_ the set of all nonnegative integer numbers, *∅* the empty set, *⌐* the logical negation operator, |*A*| the cardinality of the given set *A*, where |**N**| = *ℵ*_0_, **P**(*A*) the family of all the subsets of a given set *A*, and IDENT the set of all identifiers over a certain alphabet.

*Multiset M* over a nonempty set *S* is a function *m* : *S* → **N**_0_. The nonnegative number *m*(*a*) ∈ **N**_0_, where *a* ∈ *S*, denotes the number of occurrences of the element *a* in the multiset *m*. The multiset *m* is usually represented by the formal sum ∑_*a*∈*S*_ *m*(*a*)′*a*. *S*_MS_ denotes the set of all nonempty multisets over the set *S* and *S*_MSE_ denotes the set of all multisets over the set *S*.

Let *A* be a nonempty set. By the (nonempty finite)* sequence σ* over the set *A* a function *σ* can be understood, *σ* : {1,2,…, *n*} → *A*, where *n* ∈ **N**. The function *ε* : *∅* → *A* is called the* empty sequence* over the set *A*. The sequence *σ* : {1,2,…, *n*} → *A* is usually represented by the notation *σ* = 〈*a*_1_, *a*_2_,…, *a*_*n*_〉 or $\sigma =\begin{array}{cccc} a_{1} & a_{2} & \cdots & a_{n} \end{array}$ of the elements of the set *A*, where *a*_*i*_ = *σ*(*i*) for 1 ≤ *i* ≤ *n*. Empty sequence *ε* : *∅* → *A* over the set *A* is usually represented by the notation *ε* = 〈〉. The set of all * finite nonempty* sequences over the set *A* is denoted by the notation *A*_SQ_ and the set of all* finite* (and possible empty) sequences over the set *A* by the notation *A*_SQE_. The* length of the sequence σ* = 〈*a*_1_, *a*_2_,…, *a*_*n*_〉, where *n* ∈ **N**, is equal to the natural number *n* and the length of the empty sequence *ε* is equal to the number 0. The length of the sequence *σ* is represented by the notation length(*σ*). The function elem : *A*_SQE_ → **P**(*A*), such that elem(*σ*) = {*a*∣∃ *i* : *a* = *σ*(*i*),  where  1 ≤ *i* ≤ length(*σ*)}, elem(*ε*) = *∅*, assigns to each sequence *σ* ∈ *A*_SQE_ the set of all of its* elements*.

Let *A* = {*a*_1_, *a*_2_,…, *a*_*n*_}, where *n* ∈ **N**, be a finite nonempty set.* Vector V* is a function *V* : *A* → **N**_0_. We will denote a vector *V* by the statement *V* = (*V*(1), *V*(2),…, *V*(*n*)).

Let **V** = {(*V*(1), *V*(2),…, *V*(*n*))∣*V*(*i*) ∈ **N**_0_  for  1 ≤ *i* ≤ *n*} and **W** = {(*W*(1), *W*(2),…, *W*(*m*))∣*W*(*j*) ∈ **N**_0_  for  1 ≤ *j* ≤ *m*}. Then

(1) V⊗W=V1,…,Vn,W1,…,Wm ∣ Vi∈N0  for  1≤i≤n,  Wj∈N0  for  1≤j≤m,if  V≠∅∧W≠∅,=V,if  V≠∅∧W=∅,=W,if  V=∅∧W≠∅,=∅,if  V=∅∧W=∅.

It is clear that the operation ⊗ is associative (i.e., (**V** ⊗ **W**) ⊗ **U** = **V** ⊗ (**W** ⊗ **U**)).

A* semigroup *(*S*, *⚪*) is a set *S* together with an associative binary operation *⚪* : *S* × *S* → *S*. A* monoid *(*M*, *⚪*, *u*) is a semigroup (*M*, *⚪*) with an element *u* ∈ *M* such that ∀*x* ∈ *M* : *u* *⚪* *x* = *x* = *x* *⚪* *u*.

A* net* is an ordered triple *NET* = (*P*, *T*, *A*), where

- *P*  is a finite nonempty set of* places*,
- *T* is a finite set of* transitions*, *P*∩*T* = *∅*,
- *A* is a finite set of* arcs*, *A*⊆(*P* × *T*)∪(*T* × *P*).

A given net is then described with a bipartite graph containing a finite nonempty set *P* of places used for expressing conditions of a modeled system (we usually use circles for their representation), a finite set *T* of transitions describing changes in the modeled system (we usually draw them in the form of rectangles) and a finite set *A* of arcs being principally oriented while connecting a place with a transition or a transition with a place and we usually draw them as lines with arrows. We say then a net (*P*, *T*, *A*) is* connected* iff it is not composed of two or more disjoint and nonempty nets.

Some commonly used notations for the nets are •*y* = {*x*∣(*x*, *y*) ∈ *A*} for the* preset* and *y*• = {*x*∣(*y*, *x*) ∈ *A*} for the* postset* of a net element *y* (i.e., place or transition).

A* SNT net* (*single-number tokens net*) is an ordered 5-tuple *SNTN* = (*P*, *T*, *A*, *AF*, *TP*), where

- (*P*, *T*, *A*) is a connected net, ∀*p* ∈ *P* : *›p*⊆*›p*,
- *AF* is the arc function, *AF* : (*P* × *T*)∪(*T* × *P*) → (**N**_0_)_SQE_, *AF*(*x*, *y*) ∈ (**N**_0_)_SQ_ iff (*x*, *y*) ∈ *A*, *AF*(*x*, *y*) = 〈〉 iff (*x*, *y*) ∉ *A*, where *x*, *y* ∈ *P* ∪ *T*,
- ∀*t* ∈ *T*  ∃*k* ∈ **N**  ∀*p* ∈ (•*t* ∪ *t*•): length(*AF*(*p*, *t*)) = length(*AF*(*t*, *p*)) = *k*,
- *TP* is the transition priority function, *TP* : *T* → **N**.

The arc function *AF* of a given SNT net assigns each arc with a (nonempty) sequence of the nonnegative integer numbers expressing a removed or added token from or to the place associated with that arc when executing a particular transition. There must be the only one length *k*  (*k* ∈ **N**) of all the values of the arc function *AF* (i.e., for the sequences of nonnegative integer numbers) for the given transition *t* and all of its associated input and output arcs. Transition priority function *TP* of a given SNT net assigns with each transition the natural number value expressing its priority (transition priority function has the default value of 1 if it is not explicitly indicated in the SNT net diagram).

Let *SNTN* = (*P*, *T*, *A*, *AF*, *TP*) be a SNT net and *p* ∈ *P* a place. We will define the sets *›p* ∈ **P**(**N**) of the* place input tokens* and *p›* ∈ **P**(**N**) of the* place output tokens* by the following way:

 
*›p*=_def_{elem(*AF*(*t*, *p*))∣*t* ∈ •*p*}, if •*p* ≠ *∅*,
 
*›p*=_def_ *p›*, if (•*p* = *∅*)∧(*p*•≠*∅*),
 
*›p*=_def_ **N**, if (•*p* = *∅*)∧(*p*• = *∅*),
 
*p›*=_def_{elem(*AF*(*p*, *t*))∣*t* ∈ *p*•}, if *p*•≠*∅*,
 
*p›*=_def_ *›p*, if (*p*• = *∅*)∧(•*p* ≠ *∅*),
 
*p›*=_def_ **N**, if (•*p* = *∅*)∧(*p*• = *∅*).

Then the condition *›p*⊆*›p* must be true for all the places *p* of the given SNT net* SNTN*.

*Marking M* of the SNT net* SNTN* is a mapping *M* : *P* → (**N**_0_)_MSE_ such that ∀*p* ∈ *P*  ∀*e* ∈ *M*(*p*) : *e* ∈ *p›* (i.e., every token *e* represents the chosen single nonnegative integer number and every token *e* that is associated with the place *p* in the marking *M* must be an element of the set *p›* of the place output tokens). Marking *M* of a given SNT net then assigns each place with the multiset over the set **N**_0_ of all the nonnegative integer numbers and we use the form of 〈*m*〉 for the single token representation in the net diagrams, where *m* ∈ **N**_0_. Marking *M* of the SNT net then expresses the current status of a process that is modeled by the given SNT net. If *P* = {P1, P2,…, P*n*}, where *n* ∈ **N**, then marking *M* of given SNT net can be written as a |*P*|-vector *M* = (*M*(P1), *M*(P2),…, *M*(P*n*)).

A* SNT Petri net* is an ordered couple *SNTPN* = (*SNTN*, *M*_0_), where *SNTN* = (*P*, *T*, *A*, *AF*, *TP*) is a SNT net and *M*_0_ : *P* → (**N**_0_)_MSE_ is an initial marking of the net* SNTN*. We will then also denote a SNT Petri net by an ordered 6-tuple *SNTPN* = (*P*, *T*, *A*, *AF*, *TP*, *M*_0_).

Transition *t* ∈ *T* is* enabled* in the marking *M* of the SNT Petri net* SNTPN* if ∃ind ∈ *N*  ∀*p* ∈ •*t*  ∃*e* ∈ *M*(*p*) : *e* = *AF*(*p*, *t*)(ind) and we denote that fact symbolically in the form of *t* en  *M*.* Firing of the transition t* ∈ *T* results in changing the marking *M* into the marking *M*′, where ∀*p* ∈ *P* : *M*′(*p*) = (*M*(*p*)∖{*AF*(*p*, *t*)(ind)}) ∪ {*AF*(*t*, *p*)(ind)}, that is, denoted by *M*[*t*〉*M*′. Marking *M*_*n*+1_ is* reachable* from the marking *M*_1_ iff there exists a finite sequence $\sigma =\begin{array}{cccc} t_{1} & t_{2} & \cdots & t_{n} \end{array}$ of the transitions, where *t*_1_, *t*_2_,…, *t*_*n*_ ∈ *T*, *n* ∈ **N**, such that $\begin{array}{cccccc} M_{1}[t_{1}\rangle & M_{2}[t_{2}\rangle & M_{3} & \cdots & M_{n}[t_{n}\rangle & M_{n+1} \end{array}$. The set of all the markings reachable from the marking *M* will be denoted by the symbol [*M*〉. The set of all finite and infinite transition sequences that are reachable from the marking *M* will be denoted by the symbol [*M*⟫; that is, $[M⟫=\left\{\begin{array}{cccc} t_{1} & t_{2} & \cdots & t_{n} \end{array}|\exists M_{n+1}\in [M\rangle :M[\begin{array}{cccc} t_{1} & t_{2} & \cdots & t_{n} \end{array}\rangle M_{n+1},n\in N\right\}$.

**Figure 1:**
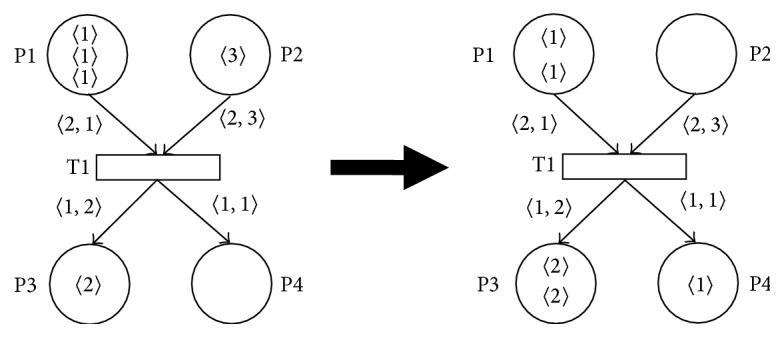
Firing of transition in SNT Petri net *N*1.

Figure 1 illustrates the SNT Petri net *N*1 = (*P*, *T*, *A*, *AF*, *TP*, *M*_0_), where

- *P* = {P1, P2, P3, P4},
- *T* = {T1},
- *A* = {(P1, T1), (P2, T1), (T1, P3), (T1, P4)},
- *AF* = {((P1, T1), 〈2, 1〉), ((P2, T1), 〈2, 3〉), ((T1, P3), 〈1, 2〉), ((T1, P4), 〈1, 1〉)},
- *TP* = {(T1, 1)},
- *M*_0_ = (3′1,1′3,1′2, *∅*).

It can be seen that ∀*t* ∈ *T*  ∃*k* ∈ **N**  ∀*p* ∈ (•*t* ∪ *t*•): length(*AF*(*p*, *t*)) = length(*AF*(*t*, *p*)) = *k* for the net *N*1, because for the only one transition T1 ∈ *T*  ∃2 ∈ **N**  ∀*p* ∈ (•T1 ∪ T1•): length(*AF*(*p*, *t*)) = length(*AF*(*t*, *p*)) = 2; that is, for any given transition *t* ∈ *T* and for all its input arcs (*p*, *t*) and all its output arcs (*t*, *p*) must be all the values of the arc functions *AF*(*p*, *t*) and *AF*(*t*, *p*) the sequences with the equal length *k*.

We can also determine that P1*›* = {1,2} (because P1•≠*∅* and P1*›* = {elem(*AF*(P1, T1))} = {1,2}), *›*P1 = {1,2} (because (•P1 = *∅*)∧(P1•≠*∅*), *›*P1 = P1*›*), P2*›* = {2,3} = *›*P2,  P3*›* = {1,2} = *›*P3, and P4*›* = {1} = *›*P4. We can also verify that the condition ∀*p* ∈ *P*  ∀*e* ∈ *M*_0_(*p*) : *e* ∈ *p›* is fully satisfied for the net *N*1.

Transition T1 is enabled in the marking *M*_0_ of the SNT Petri net *N*1 because ∃ind ∈ *N*  ∀*p*  ∈  •T1  ∃*e* ∈ *M*(*p*) : *e* = *AF*(*p*, T1)(ind), where ind = 2, •T1 = {P1, P2}, *AF*(P1, T1)(2) = 1 ∈ 2′1  +  1′4 = *M*_0_(P1), and *AF*(P2, T1)(2) = 3 ∈ 1′3 = *M*_0_(P2). Firing of the transition T1 results in changing of the net marking *M*_0_ into the marking *M*_1_, *M*_0_[T1〉*M*_1_, where *M*_1_ = (1′1 + 1′4, *∅*, 1′2 + 1′5, 1′1), because we will remove the token 〈1〉 from the place P1 and the token 〈3〉 from the place P2 and then we will add the token 〈2〉 (i.e., *AF*(T1, P3)(2)) into the place P3 and the token 〈1〉 (i.e., *AF*(T1, P4)(2)) into the place P4 according to the firing of the transition general rule.

When enabling individual transitions of a given SNT Petri net so called* conflicts* can originate in its certain markings (or conflict transitions). At the enabling of transitions *t*_1_ and *t*_2_ of the given SNT Petri net in its marking *M*, where *t*_1_, *t*_2_ ∈ *T*, the conflict occurs, if ((•*t*_1_∩•*t*_2_ ≠ *∅*)∧(*t*_1_  en  *M*)  ∧  (*t*_2_  en  *M*)∧*⌐*({*t*_1_, *t*_2_}  en  *M*)). The term of the conflict transitions can be obviously easily generalized for the case of the finite set *t*_1_, *t*_2_,…, *t*_*n*_  (*n* ∈ **N**) of transitions of a given SNT Petri net.

**Figure 2:**
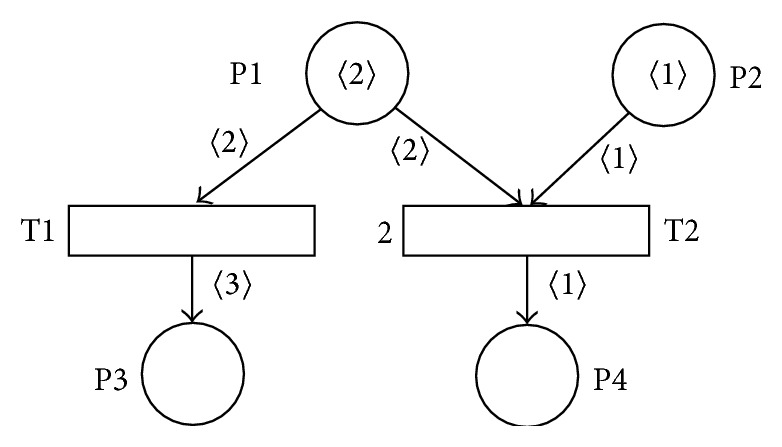
Conflict transitions in SNT Petri net *N*2.

A typical example of conflict transitions in the particular marking of the SNT Petri net *N*2 is shown in Figure 2. The transitions T1 and T2 have the common input place P1; both are enabled but not enabled in parallel. When solving such transitions conflicts we will therefore follow the rule which determines, informally said, from the set of conflict transitions the one which will be enabled, whose value of the transition priority function *TP* is the highest. If such transition from the set of conflict transitions does not exist, the given conflict would have to be solved by other means. Our studied example will be then on the basis of that rule the transition T2 enabled (because *TP*(T1) = 1 and *TP*(T2) = 2).

A* SNT process net* (PN) is an ordered 9-tuple *PN* = (*P*, *T*, *A*, *AF*, *TP*, *RP*, *IP*, *OP*, **R** **M**_**s**_), where

- (*P*, *T*, *A*, *AF*, *TP*) is a* connected SNT net*,
- *RP* is a finite set of* resource places*, *RP* ⊂ *P*,
- the* input place IP* is the only one place *IP* ∈ (*P*∖*RP*) such that •*IP* = *∅*,
- the* output place OP* is the only one place *OP* ∈ (*P*∖*RP*) such that *OP*• = *∅*,
- **R** **M**_**s**_ is the set of all the allowed* resource static markings* (see below).

The finite set of the resource places *RP* is used for expressing the conditions of a modeled system containing some initial resources and we use circles with the double line for their representation. The input place *IP* is the only one place from the set *P*∖*RP* of the nonresource places that has no input transition and similarly the output place *OP* is the only one place from the set *P*∖*RP* of the nonresource places of the SNT process net that has no output transition. The class of all the SNT process nets will be denoted by the symbol PNET.

*Marking M* of the SNT process net *PN* is again a mapping *M* : *P* → (**N**_0_)_MSE_ such that ∀*p* ∈ *P*  ∀*e* ∈ *M*(*p*) : *e* ∈ *p›*. If *RP* = {R1, R2,…, R*m*}, where *m* ∈ **N**, *P*∖*RP* = {IP, P1, P2,…, P*n*, OP}, where *n* ∈ **N**, then the marking *M* can be written as a |*P*|-vector *M* = (*M*(IP), *M*(P1), *M*(P2),…, *M*(P*n*), *M*(R1),  *M*(R2),…, *M*(R*m*), *M*(OP)).

*Static marking M*_*s*_ of the SNT process net *PN* is a marking *M*_*s*_ such that ∀*p* ∉ *RP* : *M*_*s*_(*p*) = *∅*; ∀*t* ∈ *T* : *⌐*(*t*  en  *M*_*s*_); that is, informally said, in the static marking *M*_*s*_ the tokens are allowed in the chosen resource places only and no transition is enabled in this static marking *M*_*s*_. Static marking *M*_*s*_ of the SNT process net *PN* can then be written as a |*P*|-vector *M*_*s*_ = (*∅*, *∅*, *∅*,…, *∅*, *M*_*s*_(R1), *M*_*s*_(R2),…, *M*_*s*_(R*m*), *∅*). The set of all the allowed static markings *M*_*s*_ of the SNT process net *PN* we will denote by the symbol **M**_**s**_.

If *M*_*s*_ is the given static marking of the SNT process net *PN*, then by the symbol *RM*_*s*_ we denote the* resource static marking* associated with the static marking *M*_*s*_, that is, the function *RM*_*s*_ : *RP* → (**N**_0_)_MSE_, where ∀*p* ∈ *RP* : *RM*_*s*_(*p*) = *M*_*s*_(*p*) (i.e., informally said, resource static marking *RM*_*s*_ associated with the static marking *M*_*s*_ of the SNT process net *PN* is the vector containing only the resource places markings of the static marking *M*_*s*_ and the static marking *M*_*s*_ of all the remaining places of the net *PN* is then equal to the empty set *∅*). If *RP* = {R1, R2,…, R*m*}, where *m* ∈ **N**, then the resource static marking *RM*_*s*_ of the SNT process net *PN* can be written as a |*RP*| vector *RM*_*s*_ = (*RM*_*s*_(R1), *RM*_*s*_(R2),…, *RM*_*s*_(R*m*)). The set of all the allowed resource static markings *RM*_*s*_ associated with the set **M**_**s**_ of all allowed static markings *M*_*s*_ of the SNT process net *PN* will be denoted by the symbol **R** **M**_**s**_.

*Input marking M*_*i*_ of the SNT process net *PN* is a marking *M*_*i*_ such that *M*_*i*_(*IP*) ≠ *∅*, *M*_*i*_(*OP*) = *∅*, ∃*M*_*s*_ ∈ **M**_**s**_  ∀*p* ∈ (*P*∖{IP, OP}) : *M*_*i*_(*p*) = *M*_*s*_(*p*); that is, informally said, in the input marking *M*_*i*_ the input place *IP* must contain at least one token, the output place *OP* must not contain any token, and the marking *M*_*i*_ of the remaining places corresponds with the chosen static marking *M*_*s*_ from the set of all the allowed static markings **M**_**s**_. We will denote this chosen static marking *M*_*s*_ ∈ **M**_**s**_ associated with the given input marking *M*_*i*_ by the symbol [*M*_*s*_]_*i*_. Input marking *M*_*i*_ of the process net *PN* can then be written as a |*P*|-vector *M*_*i*_ = (*M*_*i*_(*IP*), *∅*, *∅*,…, *∅*, [*M*_*s*_]_*i*_(R1), [*M*_*s*_]_*i*_(R2),…, [*M*_*s*_]_*i*_(R*m*), *∅*).

*Output (exit) marking M*_*x*_ reachable from the input marking *M*_*i*_ of the SNT process net *PN* is a marking *M*_*x*_ such that *M*_*x*_(*IP*) = *∅*, *M*_*x*_ ∈ [*M*_*i*_〉, |*M*_*x*_(*OP*)| = |*M*_*i*_(*IP*)|, and ∃*M*_*s*_′ ∈ **M**_**s**_  ∀*p* ∈ (*P*∖{IP, OP}) : *M*_*x*_(*p*) = *M*_*s*_′(*p*); that is, informally said, in the output marking *M*_*x*_ the input place *IP* must not contain any token, the output marking *M*_*x*_ is reachable from the input marking *M*_*i*_, the output place *OP* must contain the same number of tokens in the output marking *M*_*x*_ as the input place *IP* in the input marking *M*_*i*_ and the markings of the remaining places correspond with the chosen static marking *M*_*s*_′ from the set of allowed static markings **M**_**s**_. We will denote this chosen static marking *M*_*s*_′ ∈ **M**_**s**_ associated with the given output marking *M*_*x*_ by the symbol [*M*_*s*_′]_*x*_. Output marking *M*_*x*_ of the SNT process net *PN* can then be written as a |*P*|-vector *M*_*x*_ = (*∅*, *∅*, *∅*,…, *∅*, [*M*_*s*_′]_*x*_(R1), [*M*_*s*_′]_*x*_(R2),…, [*M*_*s*_′]_*x*_(R*m*), *M*_*x*_(OP)). The set of all the output markings *M*_*x*_ that are reachable from the input marking *M*_*i*_ of the SNT process net *PN* will be denoted by the symbol [*M*_*i*_〉_*x*_.

**Figure 3:**
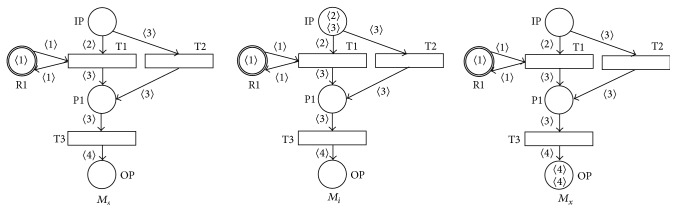
Static, input, and output markings of the SNT process net *N*3.

Figure 3 illustrates the SNT process net *N*3, where *RP* = {R1} is the only one resource place of the net, IP is the input place *IP*, OP is the output place *OP*, and the set **R** **M**_**s**_ of all its allowed resource static markings **R** **M**_**s**_ = {(1′1)}, in its static *M*_*s*_, input *M*_*i*_, and output *M*_*x*_ markings (note the resource place R1 with the initial token in the static marking *M*_*s*_) where no transition must be enabled in (any) static marking *M*_*s*_.

A* SNT process Petri net* is an ordered couple *PPN* = (*PN*, *M*_*i*_), where *PN* = (*P*, *T*, *A*, *AF*, *TP*, *RP*, *IP*, *OP*, **R** **M**_**s**_) is a SNT process net and *M*_*i*_ is an input marking of the *PN*. We will then also denote a SNT process Petri net by an ordered 10-tuple *PPN* = (*P*, *T*, *A*, *AF*, *TP*, *RP*, *IP*, *OP*, **R** **M**_**s**_, *M*_*i*_).

A* SNT agent net* (AN) is an ordered 10-tuple *AN* = (*P*, *T*, *A*, *AF*, *TP*, *RP*, *IP*, *OP*, **R** **M**_**s**_, *OT*), where

- (*P*, *T*, *A*, *AF*, *TP*) is a* connected SNT net*,
- *RP* is a finite set of* resource places*, *RP* ⊂ *P*,
- the* input place IP* is the only one place *IP* ∈ (*P*∖*RP*) such that •*IP* = *∅*,
- the* output place OP* is the selected place *OP* ∈ (*P*∖*RP*) such that *OP*• = {*OT*},
- **R** **M**_**s**_ is the set of all the allowed* resource static markings*,
- the* output transition OT* is the selected transition *OT* ∈ *T* such that •*OT* = {*OP*}, *TP*(*OT*) = 1.

The concept of the SNT agent net such extends the concept of the SNT process net. So, informally said, the output place *OP* is the selected place of the SNT agent net that has the single output transition *OT* and the output transition *OT* is the selected transition of the SNT agent net that has the single input place *OP*; it has no one output place and its transition priority function *TP* value is always the natural number 1. The meanings of the marking *M*, the static marking *M*_*s*_, the input marking *M*_*i*_, and the output marking *M*_*x*_ of the SNT agent net *AN* are the same as in the case of the SNT process net *PN*. The class of all the SNT agent nets will be denoted by the symbol ANET.

**Figure 4:**
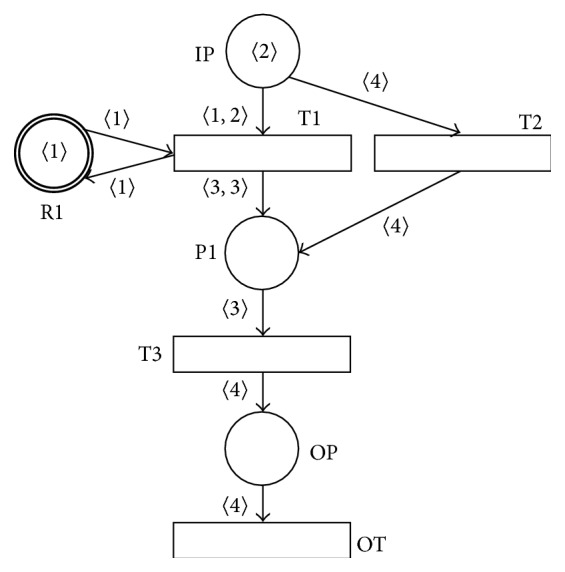
SNT agent net *N*4.

Figure 4 illustrates the SNT agent net *N*3, where *RP* = {R1} is the only one resource place, IP is the input place *IP*, OP is the output place *OP*, OT is the output transition *OT*, and the set **R** **M**_**s**_ of all its allowed resource static markings **R** **M**_**s**_ = {(1′1)}, in its input marking *M*_*i*_.

Let *AN* = (*P*, *T*, *A*, *AF*, *TP*, *RP*, *IP*, *OP*, **R** **M**_**s**_, *OT*) be a SNT agent net. The SNT process net $\underline{AN}$ associated with the SNT agent net* AN* is the 9-tuple $\underline{AN}=(\underline{P},\underline{T},\underline{A},\underline{AF},\underline{TP},\underline{RP},\underline{IP},\underline{OP},{\underline{RM}}_{\underline{s}}$), where $\underline{P}=P$, $\underline{T}=T\setminus \{OT\}$, $\underline{A}=A\setminus \{(OP,OT)\}$, $\underline{AF}=AF\setminus \{((OP,OT),k)|k\in N\}$, $\underline{TP}=TP\setminus \{(OT,1)\}$, $\underline{RP}=RP$, $\underline{IP}=IP$, $\underline{OP}=OP$, and ${\underline{RM}}_{\underline{s}}=RM_{s}$.

A* SNT agent Petri net* is an ordered couple *APN* = (*AN*, *M*_*i*_), where *AN* = (*P*, *T*, *A*, *AF*, *TP*, *RP*, *IP*, *OP*, **R** **M**_**s**_, *OT*) is a SNT agent net and *M*_*i*_ is an input marking of the *AN*. We will then also denote a SNT agent Petri net by an ordered 11-tuple *APN* = (*P*, *T*, *A*, *AF*, *TP*, *RP*, *IP*, *OP*, **R** **M**_**s**_, *OT*, *M*_*i*_). A SNT process Petri net $\underline{APN}=(\underline{AN},M_{i})$ is associated with the SNT agent Petri net *APN* = (*AN*, *M*_*i*_).

Let *PPN* = (*P*, *T*, *A*, *AF*, *TP*, *RP*, *IP*, *OP*, **R** **M**_**s**_, *M*_*i*_) be a SNT process Petri net. A place *p* ∈ *P* is said to be *k*-*bounded* iff ∃*k* ∈ **N**  ∀*M* ∈ [*M*_*i*_〉 : |*M*(*p*)| ≤ *k*. The *PPN* is said to be *k*-*bounded* iff every place of the *PPN* is *k*-bounded. We say that* PPN terminates properly* if (∀*M* ∈ [*M*_*i*_〉  ∃*M*_*x*_ ∈ [*M*_*i*_〉_*x*_ : *M*_*x*_ ∈ [*M*〉)∧(|[*M*_*i*_⟫| < *ℵ*_0_).

A SNT process net *PN* = (*P*, *T*, *A*, *AF*, *TP*, *RP*, *IP*, *OP*, **R** **M**_**s**_) is said to be* sound* iff the *PPN* = (*P*, *T*, *A*, *AF*, *TP*, *RP*, *IP*, *OP*, **R** **M**_**s**_, *M*_*i*_) is *k*-bounded and it terminates properly for its* every input marking M*_*i*_.

A SNT agent net *AN* = (*P*, *T*, *A*, *AF*, *TP*, *RP*, *IP*, *OP*, **R** **M**_**s**_, *OT*) is said to be* sound* iff the SNT process Petri net $\underline{APN}=(\underline{P},\underline{T},\underline{A},\underline{AF},\underline{TP},\underline{RP},\underline{IP},\underline{OP},{\underline{RM}}_{\underline{s}},M_{i})$ associated with the SNT agent Petri net *APN* = (*P*, *T*, *A*, *AF*, *TP*, *RP*, *IP*, *OP*, **R** **M**_**s**_, *OT*, *M*_*i*_) is sound.

From many properties of the SNT process nets, respectively, SNT agent nets, we are especially interested in their soundness (in the terminology of the programming systems this property informally means that the programming system modeled by given process net will not cause deadlock [17] and memory or device overflow).

We will start our study of the sound SNT process nets by introduction of so called* base SNT process nets* (BPN). BPN represent the class of PNs that are elementary sound (i.e., soundness of these PNs can be trivially proved).

**Figure 5:**
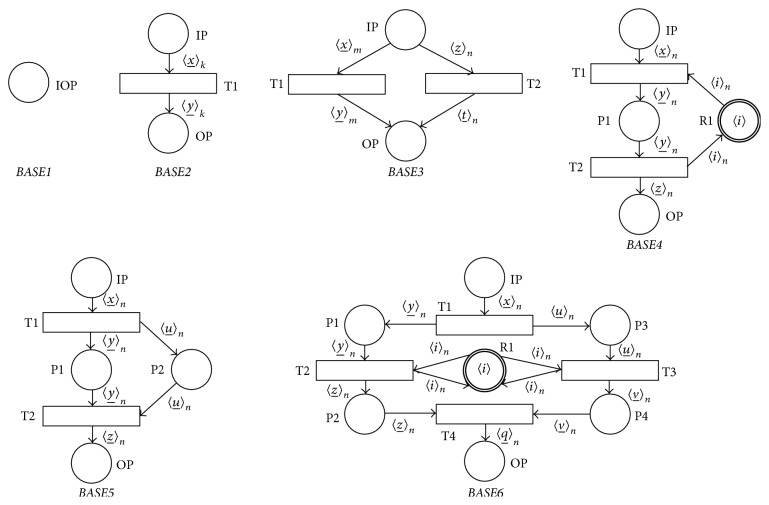
Examples of base process nets.

Figure 5 shows six simple BPNs in their static markings *M*_*s*_ ∈ **M**_**s**_ (where ${\langle \underline{x}\rangle }_{n}$ means nonempty finite sequence of the *n* nonnegative integer numbers; that is, ${\langle \underline{x}\rangle }_{n}\in {\left(N_{0}\right)}_{SQ}$, $length({\langle \underline{x}\rangle }_{n})=n$, *n* ∈ **N**, and 〈*i*〉_*n*_ means nonempty finite sequence of the *n* nonnegative integer numbers *i* ∈ **N**_0_; that is, 〈*i*〉_*n*_ ∈ (**N**_0_)_SQ_, 〈*i*〉_*n*_ = 〈*i*, *i*,…, *i*〉, and length(〈*i*〉_*n*_) = *n*, *n* ∈ **N**. The BPN* BASE1* is the simplest BPN at all and it contains only one place IOP that is simultaneously its input and output place (definition of PNs allows this case). BPN* BASE3* can be used for the modeling of the programming statement IF… THEN… ELSE…. The BPN* BASE4* then represents the model of the programming critical section, where typically |*M*_*s*_(R1)| = 1 in the resource place R1. The BPN* BASE5* represents the synchronous method called mechanism where the called method is represented by the place P2. The BPN* BASE6* can be used for the modelling of the programming barrier.

### 2.2. Place-Substitution Operator and Its Properties

The place-substitution operator PLACE-SUBST is defined for the classes of PNs and ANs and it is shown to be conditionally preserving the soundness property. The sound PNs and ANs then form a closed set (i.e., the application of this operator on any two PNs or any couple of PN and AN with the soundness property resulting in another PN or AN with the soundness property). The design of a given programming system with the support of the place-substitution operator will such typically start with the BPN* BASE1* and then it follows with the several place-substitution operations (i.e., substitution of the selected BPN over the place in the actual PN) with the support of selected BPNs that result into the complex SNT process net that models the part of the whole programming system.

**Definition 1 .**

Let *PN*1 = (*P*_1_, *T*_1_, *A*_1_, *AF*_1_, *TP*_1_, *RP*_1_, *IP*_1_, *OP*_1_, **R** **M**_**s**1_) and *PN*2 = (*P*_2_, *T*_2_, *A*_2_, *AF*_2_, *TP*_2_, *RP*_2_, *IP*_2_,  *OP*_2_, **R** **M**_**s**2_) be the PNs, and let *p* ∈ *P*_1_∖*RP*_1_ be a place of the PN *PN*1 such that

(2) ›p⊆›IP2∧OP2›⊆p›∨›p=p›=N∨›IP2=OP2›=N.

The function PLACE-SUBST: PNET × PNET → PNET of the PN *PN*2 substitution over the place *p* of the PN *PN*1 will be denoted by *PN*1.*p*〈]*PN*2. The execution of this function results in the new PN *PN* = (*P*, *T*, *A*, *AF*, *TP*, *RP*, *IP*, *OP*, **R** **M**_**s**_), where

- *P* = (*P*_1_∖{*p*}) ∪ *P*_2_,
- *T* = *T*_1_ ∪ *T*_2_,
- *A* = *A*_2_ ∪ {(*x*, *y*) ∈ *A*_1_∣(*x* ≠ *p*)∨(*y* ≠ *p*)}∪{(*x*, *IP*_2_)∣(*x*, *p*) ∈ *A*_1_}∪{(*OP*_2_, *y*)∣(*p*, *y*) ∈ *A*_1_},
- *AF* = *AF*_2_ ∪ {((*x*, *y*), *v*) ∈ *AF*_1_∣(*x* ≠ *p*)∨(*y* ≠ *p*)}∪{((*x*, *IP*_2_), *v*)∣((*x*, *p*), *v*) ∈ *AF*_1_}∪{((*OP*_2_, *y*), *v*)∣((*p*, *y*), *v*) ∈ *AF*_1_},
- *TP* = *TP*_1_ ∪ *TP*_2_,
- *RP* = *RP*_1_ ∪ *RP*_2_,
- *IP* = *IP*_1_, if *p* ≠ *IP*_1_; *IP* = *IP*_2_, if *p* = *IP*_1_,
- *OP* = *OP*_1_, if *p* ≠ *OP*_1_; *OP* = *OP*_2_, if *p* = *OP*_1_,
- **R** **M**_**s**_ = **R** **M**_**s**1_ ⊗ **R** **M**_**s**2_.

**Example 2 .**

**Figure 6:**
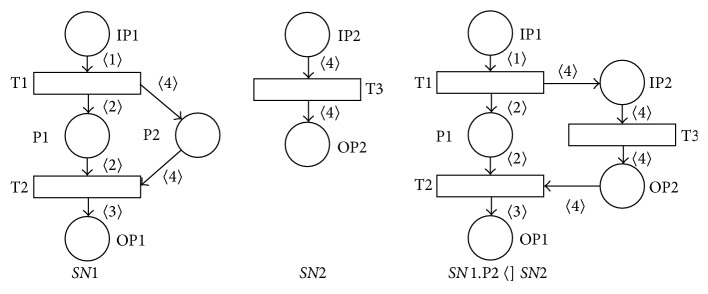
Result of place-substitution operation *SN*1.P2  〈]  *SN*2.

The result of the place-substitution operation *SN*1.P2  〈]  *SN*2 where the place *P*2 of the BPN *SN*1 (see Figure 5) was substituted by the BPN *SN*2 can be shown in Figure 6. It is clear that all the necessary requests for the place-substitution operation are satisfied in this case, that is, P2 is not a resource place of the PN *SN*1, *›*P2 = {4}⊆{4} = *›*IP2, IP2*›* = {4}⊆{4} = P2*›*. It is easy to verify that in this case the resulting PN *SN*1.P2 〈]  *SN*2 is also sound.

**Lemma 3 .**

Let *PN*1 = (*P*_1_, *T*_1_, *A*_1_, *AF*_1_, *TP*_1_, *RP*_1_, *IP*_1_, *OP*_1_, **R** **M**_**s**1_), *PN*2 = (*P*_2_, *T*_2_, *A*_2_, *AF*_2_, *TP*_2_, *RP*_2_,  *IP*_2_, *OP*_2_, **R** **M**_**s**2_), and *PN*3 = (*P*_3_, *T*_3_, *A*_3_, *AF*_3_, *TP*_3_, *RP*_3_, *IP*_3_, *OP*_3_, **R** **M**_**s**3_) be the three PNs, let *p*1 ∈ *P*_1_ be a place of the PN *PN*1 such that *p*1 ∉ *RP*_1_, ((*›p*1⊆*›IP*_2_)∧(*OP*_2_ *›*⊆*p*1*›*))∨(*›p*1 = *p*1*›* = **N**)∨(*›IP*_2_ = *OP*_2_ *›* = **N**), and let *p*2 ∈ *P*_2_ be a place of the PN PN2, such that *p*2 ∉ *RP*_2_, ((*›p*2⊆*›IP*_3_)∧(*OP*_3_ *›*⊆*p*2*›*))∨(*›p*2 = *p*2*›* = **N**)∨(*›IP*_3_ = *OP*_3_ *›* = **N**). Then

(3) PN1.p1PN2.p2PN3=PN1.p1PN2.p2PN3.

**Proof**

From Definition 1 it directly follows that *PN*1.*p*1〈](*PN*2.*p*2〈]*PN*3)    = (*PN*1.*p*1〈]*PN*2).*p*2〈]*PN*3 = (*P*, *T*, *A*, *AF*, *TP*, *RP*, *IP*, *OP*, **R** **M**_**s**_) where

- *P* = (*P*_1_∖{*p*_1_})∪(*P*_2_∖{*p*_2_}) ∪ *P*_3_,
- *T* = *T*_1_ ∪ *T*_2_ ∪ *T*_3_,
- *A* = *A*_3_ ∪ {(*x*, *y*) ∈ *A*_2_∣(*x* ≠ *p*_2_)∨(*y* ≠ *p*_2_)}∪{(*x*, *IP*_3_)∣(*x*, *p*_2_) ∈ *A*_2_}∪{(*OP*_3_, *y*)∣(*p*_2_, *y*) ∈ *A*_2_}  ∪  {(*x*, *y*) ∈ *A*_1_∣(*x* ≠ *p*_1_)∨(*y* ≠ *p*_1_)}∪{(*x*, *IP*_2_)∣(*x*, *p*_1_) ∈ *A*_1_}∪{(*OP*_2_, *y*)∣(*p*_1_, *y*) ∈ *A*_1_},
- *AF* = *AF*_3_ ∪ {((*x*, *y*), *v*) ∈ *AF*_2_∣(*x* ≠ *p*_2_)∨(*y* ≠ *p*_2_)}∪{((*x*, *IP*_2_), *v*)∣((*x*, *p*_2_), *v*) ∈ *AF*_2_}  ∪  {((*OP*_2_, *y*), *v*)∣((*p*, *y*_2_), *v*) ∈ *AF*_2_}∪{((*x*, *y*), *v*) ∈ *AF*_1_∣(*x* ≠ *p*)∨(*y* ≠ *p*)}∪{((*x*, *IP*_2_), *v*)∣((*x*, *p*), *v*)  ∈  *AF*_1_}∪{((*OP*_2_, *y*), *v*)∣((*p*, *y*), *v*) ∈ *AF*_1_},
- *TP* = *TP*_1_ ∪ *TP*_2_ ∪ *TP*_3_,
- *RP* = *RP*_1_ ∪ *RP*_2_ ∪ *RP*_3_,
- *IP* = *IP*_1_, if *p*_1_ ≠ *IP*_1_; *IP* = *IP*_2_, if (*p*_1_ = *IP*_1_)∧(*p*_2_ ≠ *IP*_2_); *IP* = *IP*_3_, if (*p*_1_ = *IP*_1_)∧(*p*_2_ = *IP*_2_),
- *OP* = *OP*_1_, if *p*_1_ ≠ *OP*_1_; *OP* = *OP*_2_, if (*p*_1_ = *OP*_1_)∧(*p*_2_ ≠ *OP*_2_); *IP* = *IP*_3_, if (*p*_1_ = *OP*_1_)∧(*p*_2_ = *OP*_2_),
- **R** **M**_**s**_ = **R** **M**_**s**1_ ⊗ **R** **M**_**s**2_ ⊗ **R** **M**_**s**3_.

**Lemma 4 .**

The triple (*PNET*,* PLACE-SUBST*,* BASE1*) is a monoid.

**Proof**

Lemma 4 is the direct consequence of Lemma 3 and it is clear that the BPN* BASE1* (see Figure 5) is the neutral element of the monoid.

**Definition 5 .**

Let *AN*1 = (*P*_1_, *T*_1_, *A*_1_, *AF*_1_, *TP*_1_, *RP*_1_, *IP*_1_, *OP*_1_, **R** **M**_**s**1_, *OT*_1_) be the AN and *PN*2 = (*P*_2_, *T*_2_, *A*_2_,  *AF*_2_, *TP*_2_, *RP*_2_, *IP*_2_, *OP*_2_, **R** **M**_**s**2_) the PNs, and let *p* ∈ *P*_1_∖*RP*_1_ be a place of the AN *AN*1 such that

(4) ›p⊆›IP2∧OP2›⊆p›∨›p=p›=N∨›IP2=OP2›=N.

The function PLACE-SUBST: ANET × PNET → ANET of the PN *PN*2 substitution over the place *p* of the AN *AN*1 will be denoted by *AN*1.*p*〈]*PN*2. The execution of this function results in the new AN *AN* = (*P*, *T*, *A*, *AF*, *TP*, *RP*, *IP*, *OP*, **R** **M**_**s**_, *OT*), where

- *P* = (*P*_1_∖{*p*}) ∪ *P*_2_,
- *T* = *T*_1_ ∪ *T*_2_,
- *A* = *A*_2_ ∪ {(*x*, *y*) ∈ *A*_1_∣(*x* ≠ *p*)∨(*y* ≠ *p*)}∪{(*x*, *IP*_2_)∣(*x*, *p*) ∈ *A*_1_}∪{(*OP*_2_, *y*)∣(*p*, *y*) ∈ *A*_1_};
- *AF* = *AF*_2_ ∪ {((*x*, *y*), *v*) ∈ *AF*_1_∣(*x* ≠ *p*)∨(*y* ≠ *p*)}∪{((*x*, *IP*_2_), *v*)∣((*x*, *p*), *v*) ∈ *AF*_1_}  ∪  {((*OP*_2_, *y*), *v*)   ∣  ((*p*, *y*), *v*) ∈ *AF*_1_},
- *TP* = *TP*_1_ ∪ *TP*_2_,
- *RP* = *RP*_1_ ∪ *RP*_2_,
- *IP* = *IP*_1_, if *p* ≠ *IP*_1_; *IP* = *IP*_2_, if *p* = *IP*_1_,
- *OP* = *OP*_1_, if *p* ≠ *OP*_1_; *OP* = *OP*_2_, if *p* = *OP*_1_,
- **R** **M**_**s**_ = **R** **M**_**s**1_ ⊗ **R** **M**_**s**2_,
- *OT* = *OT*_1_.

Definition 5 is similar to Definition 1 in the sense of overloading of the operator 〈]: PNET × PNET → PNET with the operator 〈]: ANET × PNET → ANET of the PN *PN*2 substitution over the place *p* of the AN *AN*1 which is denoted by *AN*1.*p*  〈]  *PN*2. It is easy to formulate similar lemmas like Lemmas 3 and 4 for the case of this overloaded operator 〈].

**Figure 7:**
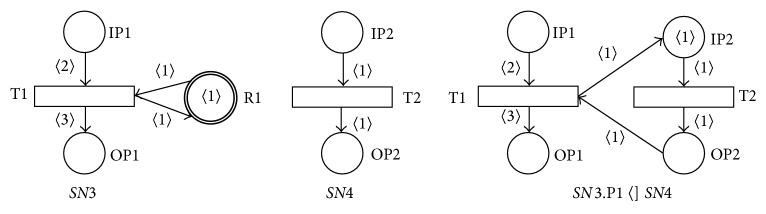
Result of place-substitution operation *SN*3.R1  〈]  *SN*4.

We will investigate the necessary conditions for the soundness of the resulting SNT process or agent net in the following paragraphs. The first condition for the soundness of the resulting net is already in Definitions 1 and 5; that is, the substituted place *p* ∈ *P* must not be a member of the set of the net resource places *RP*, *p* ∉ *RP*.  Figure 7 shows the result of the resource place R1 (that contains the token 〈1〉 in the nonempty static marking *M*_*s*_) of the sound net *SN*3 substitution by the sound net *SN*4, that is, the net *SN*3.R1  〈]  *SN*4. The resulting net then does not satisfy the necessary conditions for its static marking *M*_*s*_, because the token 〈1〉 is associated in this static marking *M*_*s*_ with the nonresource place IP2, and the transition T2 is then enabled in this static marking *M*_*s*_ which is not allowed. If we remove the token 〈1〉 from the place T2 then the resulting net is not sound again. So it is generally not possible to substitute any resource place of a given PN or AN by another PN.

**Figure 8:**
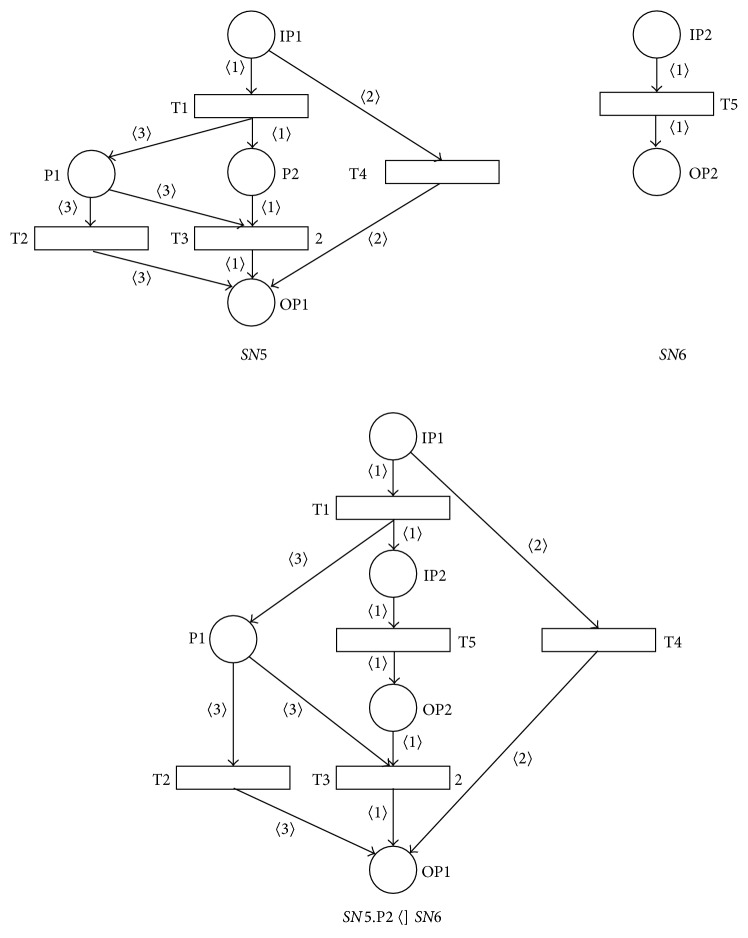
Result of place-substitution operation *SN*5.P2  〈]  *SN*6.

Figure 8 shows the result of the place P2 of the sound net *SN*5 substitution by the sound net *SN*6, that is, the PN *SN*5.P2  〈]  *SN*5, that is not sound. It can be seen for its input marking *M*_*i*_, where *M*_*i*_(*IP*1) = 1′1. In this input marking *M*_*i*_ the transition T1 is enabled and its firing results into the marking *M*_1_, where *M*_*i*_[T1〉*M*_1_. There is token 〈3〉 in the place P1 and the token 〈1〉 in the place P2 in the marking *M*_1_, and the transitions T2 and T5 are enabled in parallel in the marking *M*_1_ and after their parallel firing the PN results in the marking *M*_2_, where *M*_1_[T2  T5〉*M*_2_. In the marking *M*_2_ there will be the token 〈1〉 in the place OP2 and the token 〈3〉 in the place OP1. No transition is enabled in the nonoutput marking *M*_2_ and so the resulting net *SN*5.P2  〈]  *SN*6 is not sound.

The loss of the net soundness then follows from the existence of the arc (P1, T2) in the resulting net. The necessary condition for the soundness of the resulting net is in this case the following: ∀*t* ∈ P2•:((*q* ∈ •*t*)∧(*q* ≠ *IP*_1_))⇒(*q*• = {*t*}) and it does not hold for the place P2 of the PN  SN5, because transition T3 ∈ P2• holds that ((P1 ∈ •T3)∧(P1 ≠ IP1)) and (P1• = {T2, T3}). It is then clear that if the arc (P1, T2) is not present then the resulting net is sound.

**Figure 9:**
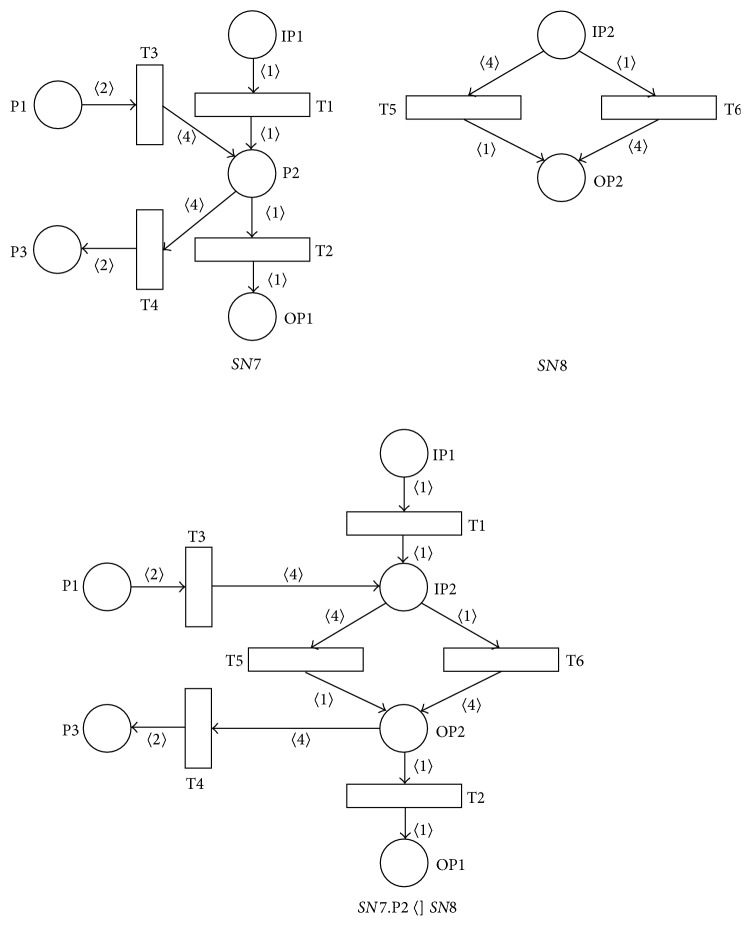
Result of place-substitution operation *SN*7.P2  〈]  *SN*8.

Figure 9 shows the result of the place *P*2 of the sound net *SN*7 substitution by the sound net *SN*8, that is, the SNT process net *SN*7.P2  〈]  *SN*8, that is again not sound. The loss of the soundness (it can be easily seen for any input marking *M*_*i*_ of the resulting PN) then follows from the fact that P2*›* = {1, 4}; that is, |*P*2*›*| > 1. The necessary condition for the soundness of the resulting PN is in this case the following: |*P*2*›*| = 1.

**Lemma 6 .**

Let *PN*1 = (*P*_1_, *T*_1_, *A*_1_, *AF*_1_, *TP*_1_, *RP*_1_, *IP*_1_, *OP*_1_, **R** **M**_**s**1_) and *PN*2 = (*P*_2_, *T*_2_, *A*_2_, *AF*_2_, *TP*_2_, *RP*_2_, *IP*_2_,  *OP*_2_, **R** **M**_**s**2_) be two sound PNs, and let *p* ∈ *P*_1_∖*RP*_1_ be a place of the *PN* *PN*1 such that ((*›p*⊆*›IP*_2_)∧(*OP*_2_ *›*⊆*p›*))∨(*›p* = *p›* = **N**)∨(*›IP*_2_ = *OP*_2_ *›* = **N**), |*p›*| = 1, ∀*t* ∈ *p*•:((*q* ∈ •*t*)∧(*q* ≠ *IP*_1_))  ⇒  (*q*• = {*t*}). Then the PN *PN*1.*p*  〈]  *PN*2 is also sound.

*Comment.* If the conditions *p* ∈ *P*_1_∖*RP*_1_, ((*›p*⊆*›IP*_2_)∧(*OP*_2_ *›*⊆*p›*))∨(*›p* = *p›* = **N**)∨(*›IP*_2_ = *OP*_2_ *›* = **N**), and |*p›*| = 1, ∀*t* ∈ *p*•:((*q* ∈ •*t*)∧(*q* ≠ *IP*_1_))⇒(*q*• = {*t*}) for the substituted place *p* ∈ *P*_1_ of the net PN1 are not satisfied the resulting net *PN*1.*p*  〈]  *PN*2 does not need to be sound generally as follows from the previous three examples.

**Proof**

It is clear for *p* = *IP*_1_ or *p* = *OP*_1_ and it follows directly from the definition of the sound PN. Let *p* ≠ *IP*_1_ and *p* ≠ *OP*_1_. Because the PN *PN*1 is sound, it holds that ∀*M*_*i*_  ∃*k* ∈ **N**  ∀*q* ∈ *P*_1_  ∀*M* ∈ [*M*_*i*_〉 : *M*(*q*) ≤ *k*. This holds also for the substituted place *p* ∈ *P*_1_. From this follows that also the place *IP*_2_ in the resulting PN is *k*-bounded because the number of tokens in any reachable marking of the process *PN*1.*p*  〈]  *PN*2 is at the highest equaling the number of tokens in the place *p*. Because the PN *PN*2 is also *k*-bounded and it terminates properly for any of its input markings *M*_*i*_, from these facts follows that also the place *OP*_2_ in the resulting PN is *k*-bounded and so the whole PN *PN*1.*p*  〈]  *PN*2 must be also *k*-bounded for any of its input markings *M*_*i*_. Similarly, both PN *PN*1 and *PN*2 terminate properly for any of its input markings *M*_*i*_. In particular for the PN *PN*1 holds true that ∀*M* ∈ [*M*_*i*_〉, *M*(*p*) ≠ *∅*, and ∃*M*_*x*_ : *M*_*x*_ ∈ [*M*〉 and for the PN *PN*2 holds true that ∀*M* ∈ [*M*_*i*_〉  ∃*M*_*x*_ : *M*_*x*_ ∈ [*M*〉. The conditions |*p›*| = 1, ∀*t* ∈ *p*•:((*q* ∈ •*t*)∧(*q* ≠ *IP*_1_))⇒(*q*• = {*t*}) are satisfied for the PN *PN*1 and from these conditions follows that |*OP*_2_ *›*| = 1, ∀*t* ∈ *OP*_2_•:((*q* ∈ •*t*)∧(*q* ≠ *IP*_1_))⇒(*q*• = {*t*}) in the resulting net *PN*1.*p*  〈]  *PN*2. Then it follows that there must exist transition *t*, *t* ∈ *OP*_2_•, and the marking *M* of the resulting net *PN*1.*p*  〈]  *PN*2 such that (*M*(*OP*_2_) ≠ *∅*)∧(*t*  en  *M*), because the PN *PN*1 terminates properly. So the whole PN *PN*1.*p*  〈]  *PN*2 must also terminate properly for any of its input markings *M*_*i*_ and it is sound.

It immediately follows from the previous lemma that the application of the operator PLACE-SUBST that satisfies all the necessary requests formulated in Lemma 6 for two given sound PNs results into another sound PN.

**Lemma 7 .**

Let *AN*1 = (*P*_1_, *T*_1_, *A*_1_, *AF*_1_, *TP*_1_, *RP*_1_, *IP*_1_, *OP*_1_, **R** **M**_**s**1_, *OT*_1_) be the sound AN and *PN*2 = (*P*_2_, *T*_2_, *A*_2_, *AF*_2_, *TP*_2_, *RP*_2_, *IP*_2_, *OP*_2_, **R** **M**_**s**2_) the sound PN, and let *p* ∈ *P*_1_∖*RP*_1_ be a place of the AN *AN*1 such that ((*›p*⊆*›IP*_2_)∧(*OP*_2_ *›*⊆*p›*))∨(*›p* = *p›* = **N**)∨(*›IP*_2_ = *OP*_2_ *›* = **N**), |*p›*| = 1, ∀*t* ∈ *p*•:((*q* ∈ •*t*)  ∧  (*q* ≠ *IP*_1_))  ⇒  (*q*• = {*t*}). Then the AN *AN*1.*p*  〈]  *PN*2 is also sound.

**Proof**

Proof directly follows from the proof of Lemma 6.

### 2.3. Asynchronous-Processing Operator and Its Properties

The asynchronous-processing operator ASYNC-PROC is the second operator defined for the class of all PNs and ANs and it is also shown to be preserving the soundness property. The sound PNs and ANs then form a closed set (i.e., the application of this operator on any couple of PN and AN or two ANs with the soundness property results in another PN or AN with the soundness property).

**Definition 8 .**

Let *PN*1 = (*P*_1_, *T*_1_, *A*_1_, *AF*_1_, *TP*_1_, *RP*_1_, *IP*_1_, *OP*_1_, **R** **M**_**s**1_) be the PN and *AN*2 = (*P*_2_, *T*_2_, *A*_2_, *AF*_2_, *TP*_2_,  *RP*_2_, *IP*_2_, *OP*_2_, **R** **M**_**s**2_, *OT*_2_) the AN, and let *t*_1_, *t*_2_,…, *t*_*n*_ ∈ *T*_1_, *n* ∈ **N**, be the transitions of the PN *PN*1. The function ASYNC-PROC: PNET × ANET → PNET of the asynchronous-processing of the AN *AN*2 from the PN *PN*1 will be denoted by *PN*1.*t*_1_.*t*_2_.⋯.*t*_*n*_  -]  *AN*2. The execution of this function results in the new PN *PN* = (*P*, *T*, *A*, *AF*, *TP*, *RP*, *IP*, *OP*, **R** **M**_**s**_), where

- *P* = *P*_1_ ∪ *P*_2_,
- *T* = *T*_1_ ∪ *T*_2_,
- *A* = *A*_1_ ∪ *A*_2_ ∪ {(*t*_1_, *IP*_2_)}∪{(*t*_2_, *IP*_2_)}∪⋯∪{(*t*_*n*_, *IP*_2_)},
- *AF* = *AF*_1_ ∪ *AF*_2_ ∪ {((*t*_1_, *IP*_2_), *k*)}∪{((*t*_2_, *IP*_2_), *m*)}∪⋯∪{((*t*_*n*_, *IP*_2_), *n*)}, where *k*, *m*, *n*,…∈*›IP*_2_,
- *TP* = *TP*_1_ ∪ *TP*_2_,
- *RP* = *RP*_1_ ∪ *RP*_2_,
- *IP* = *IP*_1_,
- *OP* = *OP*_1_,
- **R** **M**_**s**_ = **R** **M**_**s**1_ ⊗ **R** **M**_**s**2_.

**Example 9 .**

**Figure 10:**
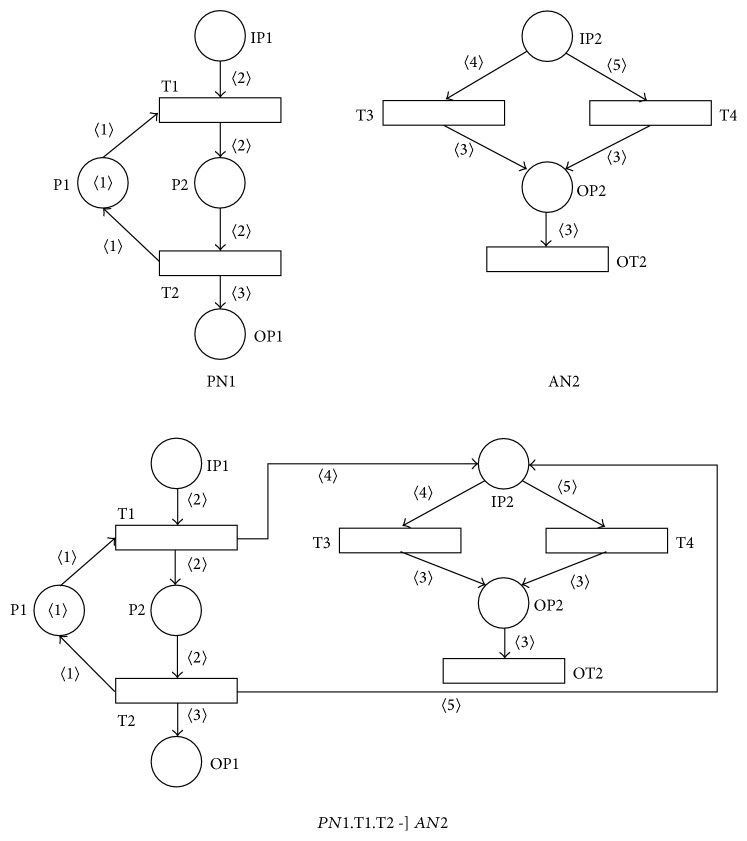
Result of asynchronous-processing operation *PN*1.T1.T2  -]  *AN*2.

The result of the asynchronous-processing operation *PN*1.T1.T2  -]  *AN*2 where both PN *PN*1 and AN *AN*2 are sound can be shown in Figure 10. It is clear that all the necessary requests for the asynchronous-processing operation are satisfied in this case. It is also easy to verify that in this case the resulting PN  *PN*1.T1.T2  -]  *AN*2 is also sound.

**Lemma 10 .**

Let *PN*1 = (*P*_1_, *T*_1_, *A*_1_, *AF*_1_, *TP*_1_, *RP*_1_, *IP*_1_, *OP*_1_, **R** **M**_**s**1_) is a PN, *AN*2 = (*P*_2_, *T*_2_, *A*_2_, *AF*_2_, *TP*_2_, *RP*_2_,  *IP*_2_, *OP*_2_, **R** **M**_**s**2_, *OT*_2_) and *AN*3 = (*P*_3_, *T*_3_, *A*_3_, *AF*_3_, *TP*_3_, *RP*_3_, *IP*_3_, *OP*_3_, **R** **M**_**s**3_, *OT*_3_) are two ANs, let *t*11, *t*21,…, *tn*1 ∈ *T*_1_ are the transition of the PN *PN*1, *n* ∈ **N**, and *t*12, *t*22,…, *tm*2 ∈ *T*_2_, *m* ∈ **N**, are the transition of the AN *AN*2. Then

(5) PN1.t11⋯tn1-AN2.t12⋯tm2-AN3=PN1.t11⋯tn1-PN2.t12⋯tm2-PN3.

**Proof**

The proof is clear and follows directly from Definition 8.

**Lemma 11 .**

The couple (*PNET* ∪ *ANET*, *ASYNC*-*PROC*) is a semigroup.

**Proof**

Lemma 11 is the direct consequence of Lemma 10.

**Definition 12 .**

Let *AN*1 = (*P*_1_, *T*_1_, *A*_1_, *AF*_1_, *TP*_1_, *RP*_1_, *IP*_1_, *OP*_1_, **R** **M**_**s**1_, *OT*_1_) and *AN*2 = (*P*_2_, *T*_2_, *A*_2_, *AF*_2_, *TP*_2_,  *RP*_2_, *IP*_2_, *OP*_2_, **R** **M**_**s**2_, *OT*_2_) be the ANs, and let *t*_1_, *t*_2_,…, *t*_*n*_ ∈ *T*_1_, *n* ∈ **N**, be the transitions of the AN *AN*1, *AN*1∩*AN*2 = *∅*. The function ASYNC-PROC: ANET × ANET → ANET of the asynchronous-processing of the AN *AN*2 from the AN *AN*1 will be denoted by *AN*1.*t*_1_.*t*_2_.⋯.*t*_*n*_  -] AN2. The execution of this function results in the new AN *AN* = (*P*, *T*, *A*, *AF*, *TP*, *RP*, *IP*, *OP*, **R** **M**_**s**_, *OT*), where

- *P* = *P*_1_ ∪ *P*_2_,
- *T* = *T*_1_ ∪ *T*_2_,
- *A* = *A*_1_ ∪ *A*_2_ ∪ {(*t*_1_, *IP*_2_)}∪{(*t*_2_, *IP*_2_)}∪⋯∪{(*t*_*n*_, *IP*_2_)},
- *AF* = *AF*_1_ ∪ *AF*_2_ ∪ {((*t*_1_, *IP*_2_), *k*)}∪{((*t*_2_, *IP*_2_), *m*)}∪⋯∪{((*t*_*n*_, *IP*_2_), *n*)}, where *k*, *m*, *n*,…∈*›IP*_2_,
- *TP* = *TP*_1_ ∪ *TP*_2_,
- *RP* = *RP*_1_ ∪ *RP*_2_,
- *IP* = *IP*_1_,
- *OP* = *OP*_1_,
- **R** **M**_**s**_ = **R** **M**_**s**1_ ⊗ **R** **M**_**s**2_
- *OT* = *OT*_1_.

Definition 12 is similar to Definition 8 in the sense of the overloading of the operator -]: PNET × ANET → PNET with the operator -]: ANET × ANET → ANET of the AN *AN*2 asynchronous-processing from the AN *AN*1. It is easy to formulate similar lemmas like Lemmas 10 and 11 for the case of this overloaded operator -].

**Lemma 13 .**

Let *PN*1 = (*P*_1_, *T*_1_, *A*_1_, *AF*_1_, *TP*_1_, *RP*_1_, *IP*_1_, *OP*_1_, **R** **M**_**s**1_) be a sound PN and *AN*2 = (*P*_2_, *T*_2_, *A*_2_,  *AF*_2_, *TP*_2_, *RP*_2_, *IP*_2_, *OP*_2_, **R** **M**_**s**2_, *OT*_2_) a sound AN, and let *t*_1_, *t*_2_,…, *t*_*n*_ ∈ *T*_1_, *n* ∈ **N**, be the transitions of the PN *PN*1. Then the PN *PN*1.*t*_1_.*t*_2_.⋯.*t*_*n*_  -]  *AN*2 is also sound.

**Proof**

Clear. Because the PN *PN*1 is sound, it holds that ∀*M*_*i*_  ∃*k* ∈ **N**  ∀*q* ∈ *P*_1_  ∀*M* ∈ [*M*_*i*_〉 : *M*(*q*) ≤ *k*. It also holds that $\forall M_{i}\;\;\exists \begin{array}{cccc} t1 & t2 & \cdots & tm \end{array}:\begin{array}{cccccc} M_{i}[t1\rangle & M_{1}[t2\rangle & M_{2} & \cdots & [tm\rangle & M_{x} \end{array}$, *m* ∈ **N**, *t*1, *t*2,…, *tm* ∈ *T*_1_. From these facts follows that every of the transition *t*_1_, *t*_2_,…, *t*_*n*_ ∈ *T*_1_, *n* ∈ **N**, will be fired finite times in every transition sequence $\begin{array}{cccccc} M_{i}[t1\rangle & M_{1}[t2\rangle & M_{2} & \cdots & [tm\rangle & M_{x} \end{array}$. From these immediately follows that in the resulting PN *PN*1.*t*_1_.*t*_2_.⋯.*t*_*n*_  -]  *AN*2 is satisfied the condition ∀*M*_*i*_  ∃*k* ∈ **N**  ∀*M* ∈ [*M*_*i*_〉 : *M*(*IP*_2_) ≤ *k*. Because of the soundness of the AN AN2 the whole PN *PN*1.*t*_1_.*t*_2_.⋯.*t*_*n*_  -]  *AN*2 must be bounded. Because the PN *PN*1 terminates properly for any of its input markings *M*_*i*_ and also the AN *AN*2 terminates properly and their firing of the transitions are independent it is clear that also the resulting PN  *PN*1.*t*_1_.*t*_2_.⋯.*t*_*n*_  -]  *AN*2 must terminate properly for any of its input marking and it is also sound.

### 2.4. Audiovisual Lecture Room with the Automatic Editing System

**Figure 11:**
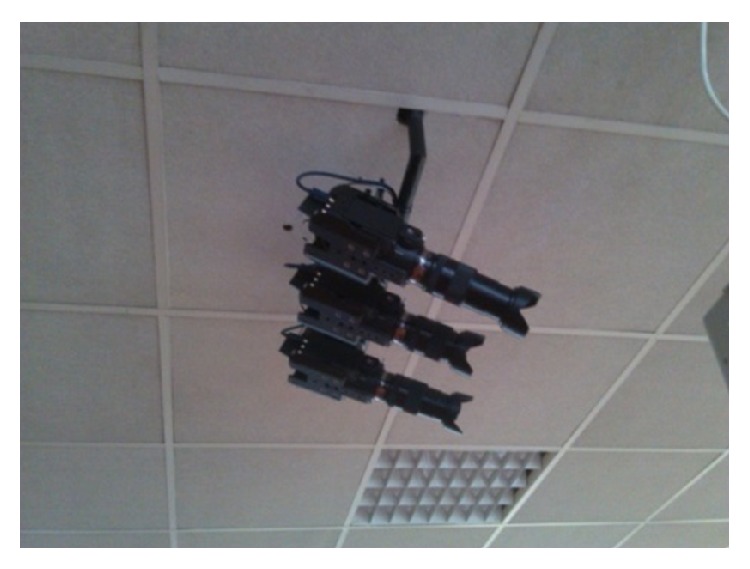
Three cameras Sony BPE NEX-FS100EK.

Within the MERLINGO project implementation the selected standard audiovisual lecture room was additionally outfitted with the automated editing system consisting of three good quality cameras* Sony BPE NEX-FS100EK* series with the* object lens E18-200 mm* to eliminate the problem of insufficient lighting in lecture rooms during recording (see Figure 11), followed by editing unit* Roland VR-5* including three spatial microphones* Roland R-26* and control system with the motion sensors of* AMX 3100* line with the converter* RS232/MiDi*. That system ensures editing of video and audio signal read by the camera and microphone in the sectors where the lecturer is moving.

Additionally, this audiovisual lecture room has been outfitted with the programming system* EduArt *enabling transfer of image (video) of the scanned person and transfer of presentation from the work station, interactive board, visualizer, and sound in the high resolution via the computer network of the sufficient bandwidth. The output is a recording viewable by the web browser. The integration of the whole unit is ensured by the control system of the AMX product line automated in such manner that the lecturer can concentrate on the context of their message and does not need to pay attention to control of presentation tools and means for the recording and transfer of the presentation into the resources of the central repository MERLINGO. There is no need for other persons to make a recording which is a cost saving feature and, additionally, the lecturer is not disturbed by them.

**Figure 12:**
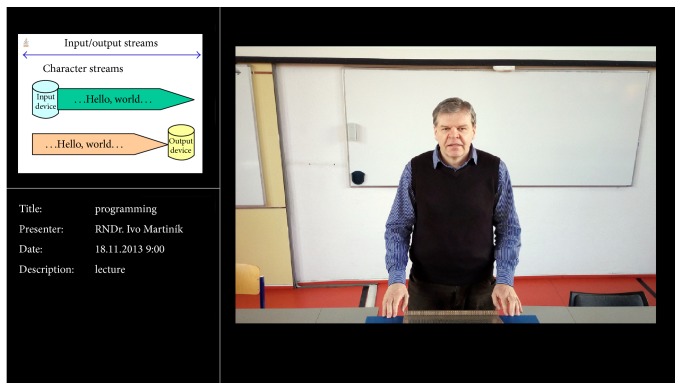
Shot of* detail-view* type in audiovisual lecture room.

**Figure 13:**
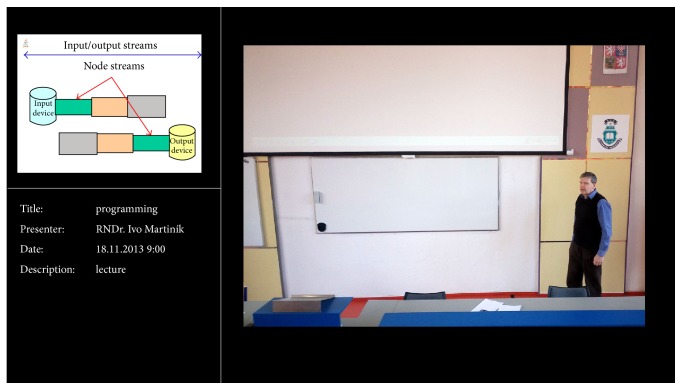
Shot of* general-view* type in audiovisual lecture room.

For the realization of the automated editing were selected and implemented three basic types of shots:

- *detail-view*: lecturer (one or more) standing by the speaker's desk; see Figure 12,
- *board-view*: lecturer (one or more) are found in the zone of the board or in the zone between the speaker's desk and board,
- *general-view*: lecturer (one or more) are found outside the shot* detail-view* or* board-view*; see Figure 13.

## 3. Results and Discussion

The SNT process and agent net formal theory have been used at the design, verification, simulation, and implementation phases of software support of the audiovisual lecture room enabling automated recordings of the presentations including automated editing.

When the speaker enters or exits one of the* general-view*,* board-view*, or* detail-view* areas the corresponding area sensor in the audiovisual lecture room sends the message describing this event. We will model these events by the tokens 〈1〉–〈6〉 in the resulting PN* RNET* by the following way:

$\langle 1\rangle$ entry of the speaker into the* general-view* area (from the nonscanned area of the lecture room),
$\langle 2\rangle$ exit of the speaker outside the* general-view* area (into the nonscanned area of the lecture room),
$\langle 3\rangle$ exit of the speaker outside the* general-view* area and entry of this speaker into the* board*-*view* area,
$\langle 4\rangle$ exit of the speaker outside the* board-view* area and entry of this speaker into the* general-view* area,
$\langle 5\rangle$ exit of the speaker outside the* board-view* area and entry of this speaker into the* detail-view* area,
$\langle 6\rangle$ exit of the speaker outside the* detail-view* area and entry of this speaker into the* board-view* area.

**Figure 14:**
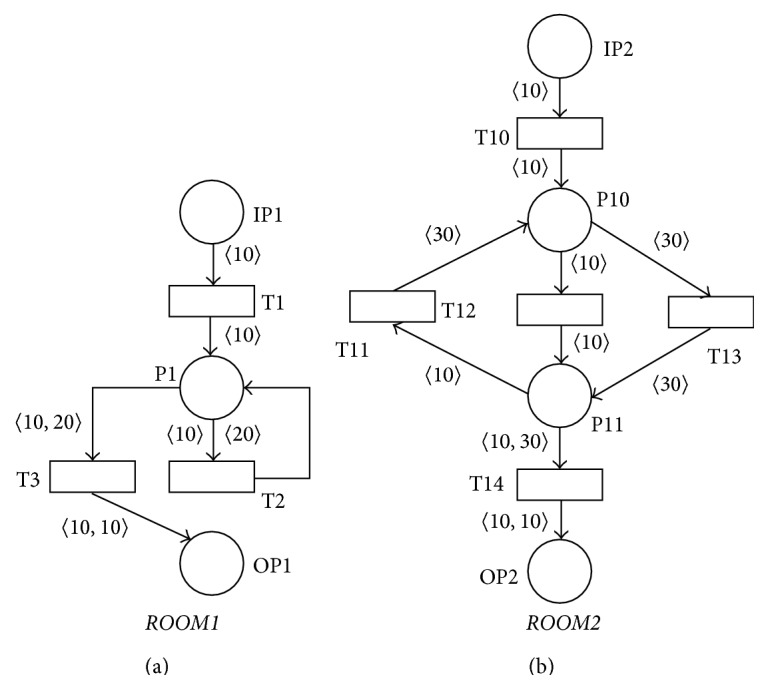
SNT process nets* ROOM1* and* ROOM2.*

The PN* ROOM1* in Figure 14(a) then models the basic functionalities of the lecture room during the presenter(s) entry or exit into or outside the* general-view* area from or into the nonscanned area of the lecture room. The individual speaker that enters into the* general-view* area is represented by the token 〈10〉 in the input place IP1 of the PN* ROOM1* in its input marking *M*_*i*_. The input place IP1 can contain in its input marking *M*_*i*_ any finite number of these tokens. The firing of the transition T1 then represents an entry of the selected speaker into the* general-view* area from the nonscanned area of the lecture room. This entry then generates the activity of the chosen* general-view* area sensor that is modeled by the message represented by the token 〈1〉 in the resulting PN. Speaker can then leave out the* general-view* area into the nonscanned area of the lecture room by the firing of the transition T3 (the message represented by the token 〈2〉 will be sent by the selected* general-view* area sensor as the consequence of this action). The transition T2 is executed when the speaker leaves out the* general-view* and then he enters into the remaining areas of the lecture room. The token 〈10〉 that represents the chosen speaker inside the lecture room will be changed into the token 〈20〉 after the firing of the transition T2. This change of the token after the firing of the transition T2 will such disable the possibility of the infinite transitions sequence $\begin{array}{ccc} T2 & T2 & \cdots \end{array}$ that can be potentially fired by every token 〈10〉 in the marking of the place P2. Every token 〈20〉 in the actual marking of the place P2 can then enable only the transition T3 that models leaving out the* general-view* area into the nonscanned area of the lecture room by the given speaker. It is clear that the PN* ROOM1* is sound for every one of its input markings *M*_*i*_.

The PN* ROOM2* in Figure 14(b) then models the basic functionalities of the lecture room during the presenter(s) entry or exit into or outside* board-view* (represented by the place P10 and output place OP2) and* detail-view* (represented by the place P11) areas. The token(s) 〈10〉 in the marking of the input place IP2 represent(s) individual speaker(s) in the* general-view* area of the lecture room. The firing of the transition T10 models the entry into the* board-view* area by the individual speaker and sending of the message represented by the token 〈3〉. The entry into the* detail-view* area by the individual speaker and sending of the message represented by the token 〈5〉 is then modeled by the firing of the transition T12. Every presenter has the possibility of revisiting the* board-view* area during its presentation (firing of the transition T11 and sending of the message 〈6〉) and returning again into the* detail-view* area (firing of the transition T13 and sending of the message 〈5〉). Every such speaker is then represented by the token 〈30〉 after the firing of the transition T11 to prevent the possibility of the infinite transitions sequence $\begin{array}{ccccc} T11 & T13 & T11 & T13 & \cdots \end{array}$. The firing of the transition T14 and sending of the message represented by the token 〈4〉 then model an entry of the selected speaker back into the* board-view* area from the* detail-view* area of the lecture room. It is again clear that the PN* ROOM2* is sound for every one of its input markings *M*_*i*_.

PN* ROOM* that models all the main functionalities of the audiovisual lecture room is then the PN* ROOM* =* ROOM1*.P1 〈]* ROOM2* that is also sound according to Lemma 6.

**Figure 15:**
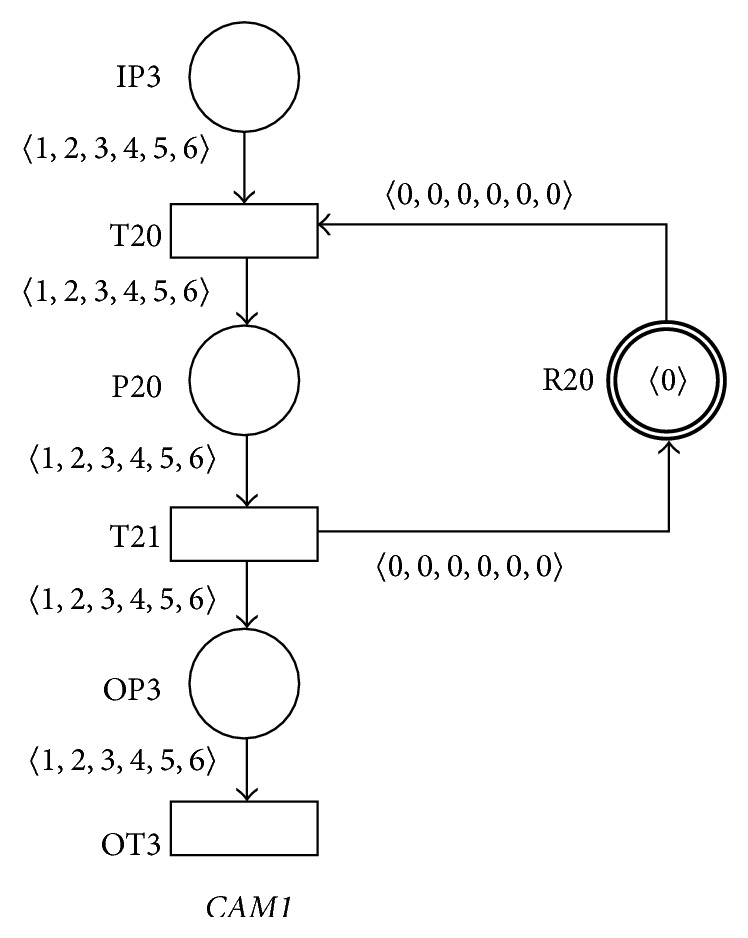
SNT agent net* CAM1.*

Design and implementation of the programming systems that accept the messages generated by the area sensors of the audiovisual lecture room (these messages are represented by the tokens 〈1〉–〈6〉 in the resulting PN) and that realize the automatic switching of the camera systems and the automatic editing functionality during recordings of presentations are represented by the AN* CAM1* in Figure 15. The input place IP3 of the AN* CAM1* accepts all the messages represented by the tokens 〈1〉–〈6〉 and generated by the area sensors of the audiovisual lecture room. Resource place R20 with the token 〈0〉 of the only one allowed static marking *M*_*s*_ of this AN (this static marking *M*_*s*_ is such the only-one element of the set **M**_**s**_ of the AN* CAM1*) represents the critical section mechanism of the whole programming system. This critical section guarantees that at most one message will be serviced by the programming system at the given time. The place P2 then represents the algorithm which realizes the automatic switching of the camera systems. The firing of the output transition OT3 then discards every accepted message from the given area sensor. It is again clear that the AN* CAM1* is sound for every one of its input markings *M*_*i*_.

**Figure 16:**
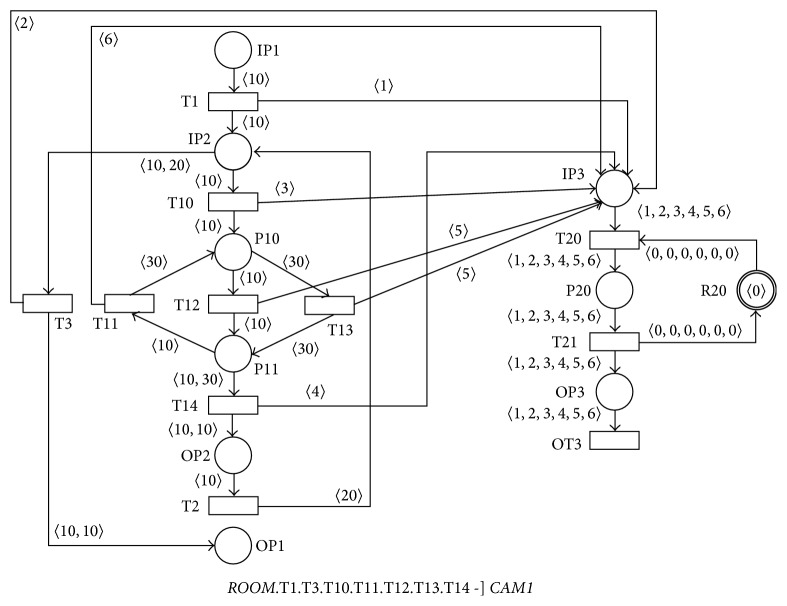
SNT process net* RNET1.*

Figure 16 shows the resulting PN* RNET1*  that is composed of the PN* ROOM* =* ROOM1*.P1 〈]* ROOM2* and the AN* CAM1* using the* ASYNC-PROC* operator such that* RNET1* =* ROOM*.T1.T3.T10.T11.T12.T13.T14 -]* CAM1*. The PN* RNET1* introduces the mechanism of the messages generated by the area sensors of the audiovisual lecture room processing (these messages are represented by the tokens 〈1〉–〈6〉 in the PN* RNET1*). The whole PN* RNET1* is again sound according to Lemma 13.

**Figure 17:**
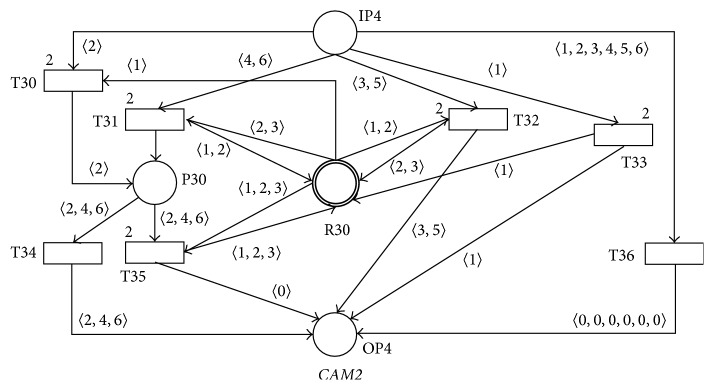
SNT process net* CAM2.*

The first inner part of the programming systems that accepts the messages generated by the area sensors of the audiovisual lecture room is modeled by the PN* CAM2* in Figure 17. Every token of the actual static marking *M*_*s*_ of the only one resource place R30 of this PN represents the speakers that are actually presenting in the* general-view*,* board-view,* or* detail-view* areas at the given time. Every (anonymous) speaker that is presenting in the* general-view*,* board-view*,* detail-view* areas, respectively, at the given time is then modeled by the tokens 〈1〉, 〈2〉, and 〈3〉, respectively, in the actual static marking *M*_*s*_ of the resource place R30. The set of all the allowed resource markings of this PN is then **R** **M**_**s**_ = {(*i*′〈1〉 + *j*′〈2〉 + *k*′〈3〉)∣*i* ∈ **N**_0_, *j* ∈ **N**_0_, *k* ∈ **N**_0_}. The functionalities of the PN* CAM2* are as in the following:

- Acceptance of the message 〈1〉 (entry of the speaker into the* general-view* area from the nonscanned area of the lecture room) results in the firing of the transition T33 and adding of the token 〈1〉 (entry of the speaker into the* general-view* area) into the resource place R30.
- Acceptance of the message 〈3〉 (exit of the speaker outside the* general-view* area and entry of this speaker into the* board-view* area) results in the firing of the transition T32, removing the token 〈1〉 (exit of the speaker outside the* general-view* area) from the resource place R30 and adding the token 〈2〉 (entry of the speaker into the* board-view* area) into the resource place R30.
- Acceptance of the message 〈5〉 (exit of the speaker outside the* board-view* area and entry of this speaker into the* detail-view* area) results in the firing of the transition T32, removing of the token 〈2〉 (exit of the speaker outside the* board-view* area) from the resource place R30, and adding of the token 〈3〉 (entry of the speaker into the* detail-view* area) into the resource place R30.
- Acceptance of the message 〈2〉 (exit of the speaker outside the* general-view* area into the nonscanned area of the lecture room) results in the firing of the transition T32 and removing of the token 〈1〉 (exit of the speaker outside the* general-view* area into the nonscanned area) from the resource place R30. If the resource place R30 still contains at least one token 〈1〉 (i.e., at least one speaker is still presented in the* general-view* area) the transition T35 then fires and the token 〈2〉 is changed into the token 〈0〉 (i.e., no camera switching will be performed). The transition T34 will fire otherwise and the camera switching can be performed.
- Acceptance of the message 〈4〉 (exit of the speaker outside the* board-view* area and entry of this speaker into the* general-view* area) results in the firing of the transition T31, removing of the token 〈2〉 (exit of the speaker outside the* board-view* area) from the resource place R30 and adding of the token 〈1〉 (entry of this speaker into the* general-view* area) into the resource place R30. If the resource place R30 still contains at least one token 〈2〉 (i.e., at least one speaker is still presented in the* board-view* area) the transition T35 then fires and the token 〈4〉 is changed into the token 〈0〉 (i.e., no camera switching will be performed). The transition T34 will fire otherwise and the camera switching can be performed.
- Acceptance of the message 〈6〉 (exit of the speaker outside the* detail-view* area and entry of this speaker into the* board-view* area) results in the firing of the transition T31, removing of the token 〈3〉 (exit of the speaker outside the* detail-view* area) from the resource place R30, and adding of the token 〈2〉 (entry of this speaker into the* board-view* area) into the resource place R30. If the resource place R30 still contains at least one token 〈3〉 (i.e., at least one speaker is still presented in the* detail-view* area) the transition T35 then fires and the token 〈6〉 is changed into the token 〈0〉 (i.e., no camera switching will be performed). The transition T34 will fire otherwise and the camera switching can be performed.
- The transition T35 will fire otherwise and no camera switching will be performed.

It is not difficult to prove that the whole PN* CAM2* is sound for every one of its input markings *M*_*i*_.

**Figure 18:**
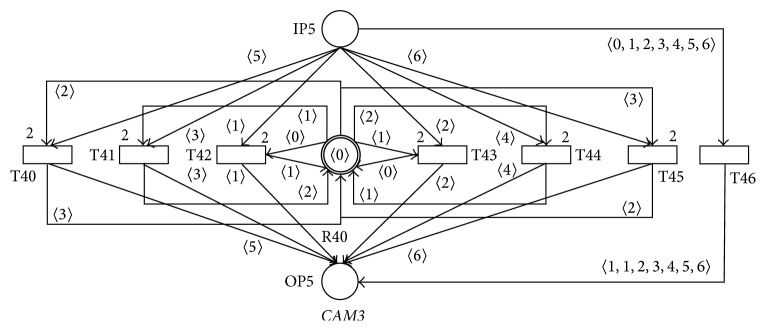
SNT process net* CAM3.*

The second inner part of the programming systems that accepts the messages generated by the area sensors of the audiovisual lecture room is modeled by the PN* CAM3* in Figure 18. Every token of the actual static marking *M*_*s*_ of the only one resource place R40 of this PN represents the actual state of the cameras for the* general-view*,* board-view*, or* detail-view* areas, such that

- the token 〈0〉 represents the state where all the three cameras are nonactive,
- the token 〈1〉 represents the state where only the shot for the* general-view* area is actively scanned and all of the remaining cameras are nonactive,
- the token 〈2〉 represents the state where only the shot for the* board-view* area is actively scanned and all of the remaining cameras are nonactive,
- the token 〈3〉 represents the state where only the shot for the* detail-view* area is actively scanned and all of the remaining cameras are nonactive.

The set of allowed resource markings of this PN is then **R** **M**_**s**_ = {(〈*k*〉)∣*k* ∈ {0,1, 2,3}}. The functionalities of the PN* CAM2* are as in the following:

- Acceptance of the message 〈1〉 (entry of the speaker into the* general-view* area from the nonscanned area of the lecture room) results in the firing of the transition T42 only if all the three cameras are in nonactive states (i.e., the token 〈0〉 is actually presented in the marking of the resource place R40) and the token 〈1〉 will be then added into the resource place R40 (i.e., only the camera for the* general-view* area is in active state and all of the remaining cameras are in nonactive states), otherwise the transition T46 will fire and the actual states of all the cameras will be unchanged,
- Acceptance of the message 〈2〉 (exit of the speaker from the* general-view* area and entry into the nonscanned area of the lecture room) results in the firing of the transition T43 if only the camera for the* general-view* area is in active state and all of the remaining cameras are in nonactive states (i.e., the token 〈1〉 is actually presented in the marking of the resource place R40) and the token 〈0〉 will be then added into the resource place R40 (i.e., all the cameras are in nonactive states), otherwise the transition T46 will fire and the actual states of all the cameras will be unchanged,
- … and similarly for the acceptance of the messages 〈3〉–〈6〉.

It is again clear that the PN* CAM3* is sound for every one of its input markings *M*_*i*_.

We will then construct the resulting PN* RNET* with the support of the PLACE-SUBST operator applications such that

(6) $$\begin{array}{c} RNET=\left(\;RNET1.P20\langle ]CAM2\;\right).OP4\langle ]CAM3, \end{array}$$

where *RNET*1 = *ROOM*.T1.T3.T10.T11.T12.T13.T14  -]* CAM1* and *ROOM* = *ROOM*1.P1  〈]  *ROOM*2; that is,

(7) RNET=ROOM1.P1ROOM2.T1.T3.T10.T11.T12.T13.T14-CAM1.P20CAM2.OP4CAM3.

The resulting PN* RNET* is also sound according to Lemmas 6 and 13.

## 4. Conclusions

Design and implementation of the programming systems which realize the automatic switching of the camera systems and the automatic editing functionality during recordings of presentations in the audiovisual lecture room involved the use of the SNT Petri process and agent nets formal theory. The simplified SNTPAN model of the given system is shown in Figures 14–18 (a more detailed model is much more complicated and its design used the theory of the timed SNTPANs [18]).

There is an opportunity to define additional operators for the class of SNTPANs and to generalize it (for instance, the SYNC-PROC composition operator that models synchronous nonrecursive calling of chosen method represented by a PN *PN*2 of some programming system from the body of another method of the same programming system represented by a PN *PN*1). Selected PN or AN then represents typically a method in the model of the programming system realized with the support of the SNTPAN formalism.

At the Faculty of Economics, VŠB-Technical University of Ostrava, the recordings of presentations were realized with the support of highly mentioned lecture room with the automated editing. The rich-media recordings were published through the* MediaInTouch* programming system that is also integrated with the LMS system* Moodle* in the present time. This integration is based on the presence of the accessibility of the* MediaInTouch* application programming interface based on the web services technology. Thus it is possible to enable (i.e., insert or share) the recordings of presentations from the* MediaInTouch* repository as the organic part of every eLearning course content in the* Moodle* environment. The recordings are such then also the parts of the study materials for the mentioned subjects in the Bachelor and Master studies in the* Moodle* environment.

The lecture room with the support of automated editing enables* all-day fully automated* realization of all presentation recordings in the present time. There is then significant financial, personal, and time cost reduction joined with the creation and management of rich-media learning objects in comparison of the usual ways of recording. There is also verifiable improvement of the study results of the students (and particularly of the students with special needs) in the context of the availability of the presentation recordings and their accessibility online or on-demand as the standard part of the virtual university resources.

Those technologies can be also crucially beneficial during qualitative extension of provided services for the students with special needs, mainly at the establishment of “barrier-free” information access to recordings of presentations adapted to needs mainly for students with locomotive, visual aural disability while using internationally valid standards.* EduArt* and* MediaInTouch* programming systems are also extensively applied at the practical application of the methodology of adaptation of existing and newly created learning objects which are adapted for students with special needs. The main results achieved in this area currently involve

- technical solution of the parallel recording of translation of the lecturer into the sign language and text form. The programming implementation of the stated feature is based on the addition of next video stream containing translation of the lecturer into sign language or text form in the resulting record and their synchronizing with performed presentation,
- the transcription of standard eLearning text study supports in the audio form and their availability obtained via podcasting as a part of the MERLINGO portal services,
- automated transcription of spoken text of the lecture recorded by the recording and assistance service into the written text.
